# Glenda Gray: overcoming obstacles, driving innovation

**DOI:** 10.2471/BLT.25.030125

**Published:** 2025-01-01

## Abstract

Glenda Gray talks to Gary Humphreys about her life and career in HIV prevention and treatment and the ongoing struggle to develop an effective vaccine.

*Q: You’re known for your work as a clinician, scientist, advocate and activist. How did those different strands come together?*

A: I grew up poor in a small mining town in apartheid South Africa, so right from the beginning I had a clear understanding of what it was like to struggle and also knew what social injustice looked like. But even though we were poor, my dad, who was a mining engineer, always stressed the importance of getting an education and insisted that my five siblings and I go to university. As for the desire to practise medicine, it was just something inside me, I think. On the activism front, I blame my eldest brother. He went to Witwatersrand University, where he became very politically aware, and he shared that awareness at the dinner table when he came home. By the time I went to medical school in 1981, I was definitely what you would call an activist. For example, I was part of a committee that supported political detainees who had been released from jail. As a medical student my role was to examine them, document their injuries, and take histories of their torture. That had a huge impact on me.

*Q: You were on course to be a paediatrician. What happened?*

A: HIV [human immunodeficiency virus] happened. By 1985, the virus was spreading across the continent. The government’s response was to blame the miners and to deport those who had come in from Malawi and Lesotho. I joined a health workers' association, focusing on AIDS [acquired immunodeficiency syndrome]-related matters, but it was at Baragwanath Hospital in Soweto, in 1988, that I really started to see cases. I was working in the ICU [intensive care unit] and more and more people were coming in with HIV-related complications. Then, in 1989, children started to show up. It seemed to happen almost overnight. By 1996, every third child in the ward had HIV. That was a turning point for me. My original vision had been to run my own ward at Baragwanath, caring for children and training the next generation of paediatricians, but as the HIV epidemic grew, I found myself drawn into research projects. In 1993, along with my colleague the obstetrician Dr James McIntyre, I established a perinatal HIV clinic at Baragwanath. It was among the first in the country to offer HIV testing and counselling for pregnant women and to conduct community outreach. It was a wonderful clinic, because it kept pregnant women and mothers with their babies together instead of separating them because of the disease. We also created a strong support network – women who had been through the process could support newly diagnosed women. It was also a great place to do research, and in 1996, the clinic evolved into the Perinatal HIV Research Unit, which was affiliated with the University of the Witwatersrand.

“We were very deliberate in pushing the boundaries.” 

*Q: Where did you initially direct your attention?*

A: One of my early studies focused on empowering women to make informed choices about how to feed their babies. It was understood by that time that the virus could be transmitted through breastmilk, and the World Health Organization (WHO) was recommending that mothers in rich countries should switch to formula. But they were recommending that women in poor countries should breastfeed, because substituting breastmilk for formula ran the risk of exposing the baby to waterborne pathogens. I felt this was deeply unfair, and in my study spoke directly to the women concerned, explaining the risks and options and telling them to make their own decisions. In Soweto, we supported women who chose formula feeding by ensuring they had access to clean water, proper feeding techniques and formula supplies. It caused quite a stir. Some people accused me of promoting formula feeding and even being on Nestlé’s payroll, which was particularly galling!

Another study we carried out was to evaluate the efficacy of short-course antiretroviral (ART) regimens in preventing mother-to-child transmission of HIV. It ran between 1996 and 2000 across multiple sites in South Africa, Tanzania and Uganda and was quite successful, laying the groundwork for subsequent studies and programmes. At one point in the trial there was discussion about the fact that some of the women were getting a placebo. We explained the reasoning to the participants, and they agreed to continue, but they made us promise that every woman in Soweto would get access to the intervention. It wasn’t easy, but we found ways even in the face of intense government denialism and obstruction.

*Q: How did you push back against that?*

A: We operated with a mix of rage and determination. For example, when the University Human Research Ethics Committee refused to approve studies for ART, we’d take women and their babies in our cars to meet the committee in person. We’d be having meetings and the room would be filled with these mothers and their babies, and they would tell their stories directly to the ethicists. We were very deliberate in pushing the boundaries to make the decision-makers see the people affected by their choices. I like to think we had an impact.

*Q: When did the government’s position begin to change on HIV?*

A: That’s a matter for debate, but it’s worth noting that as early as 1999, during the height of AIDS denialism in South Africa, the government, in collaboration with the Department of Science and Technology, the Department of Health, Eskom (South African electricity public utility) and the mining industry came together to fund the South African AIDS Vaccine Initiative (SAAVI). SAAVI entrusted the SAMRC with the task of developing an HIV vaccine, and it was the SAMRC who approached me to lead the clinical development side of the vaccine programme in 2014. I accepted.

*Q: How steep was the learning curve?*

A: It was fairly daunting, but my role as a clinician scientist gave me a foundation, and I was intrigued by the science of HIV vaccines. I became deeply invested in understanding both immunogenicity and the determinants of HIV acquisition, exploring how interventions could work to prevent infections. I also relish a challenge, I think. The organization was struggling when I took over its funding was limited, and its reputation had taken a hit, but I felt like there was a lot to be done and I threw myself into it, transforming the way we funded medical research, and expanding the research portfolio. I also believed in leading by example, so while I handled my administrative duties, I remained active as a scientist. I continued to conduct clinical trials, including the HVTN 702 (Uhambo) trial, a large-scale HIV vaccine clinical trial that was launched in 2016, and the HVTN 705 (Imbokodo) trial, which was launched in 2017 to test the efficacy of a vaccine regimen designed to provide protection against a wide range of HIV strains.

*Q: Neither of those trials proved successful. How hard was it to accept those failures?*

A: Each trial was conducted with the assumption that success was possible, even inevitable. You have to do it that way in order to fully commit yourself. So, when the trials failed, it was devastating. You pour your heart into the science, and then you have to face the community and participants to explain the results. It’s a deeply emotional and challenging experience, and it doesn’t get easier, no matter how many times it happens.

“Giving up on an HIV vaccine would mean giving up on science itself.”

*Q: What were the main lessons learned from those trials?*

A: We learned a lot. First, we saw how high the incidence rates still were, underscoring the urgency of our work. We also learned that uptake of oral pre-exposure prophylaxis is low even when it's available, with behavioural factors and preferences playing a huge role in intervention success. On the scientific side, we found that non-human primate models often don’t predict human outcomes. Also, our idea that vaccines targeting common versions of the virus would work didn’t pan out. We also learned that neutralizing antibodies – ones that directly block the virus – are absolutely necessary for an effective vaccine, but that creating a vaccine that reliably triggers strong neutralizing antibody responses is very, very difficult.

*Q: How optimistic are you that an HIV vaccine will be found?*

A: I remain hopeful, partly because we have come so far with biotechnology. Today, we have tools like crystallography and structural biology to study the structure of HIV in extraordinary detail. We have a better understanding of how antibodies interact with susceptible sites on the HIV envelope. The advent of mRNA technology has also been transformative and may help us overcome some of the barriers we’ve faced with HIV vaccine development. The progress being made with tuberculosis (TB) vaccines such as the M72 vaccine, is intriguing in this regard. At the same time, we now have game-changing tools like long-acting antiretrovirals, which can control HIV effectively if we can make them accessible.

*Q: Do long-acting antivirals make it harder to justify continued expenditure on HIV-vaccine development?*

A: That’s a fair question, but for me, giving up on an HIV vaccine would mean giving up on science itself. The lessons we’ve learned through HIV research have already contributed so much to other fields. For example, the work done by the Vaccine Research Centre to develop HIV vaccines directly informed the rapid development of COVID-19 vaccines, particularly with the fusion protein research led by Barney Graham. Without the investments in HIV science, our response to SARS-CoV-2 would have been far slower.

*Q: You were recently appointed chair of the board of directors for the Global Antibiotic Research and Development Partnership (GARDP). What will be the main focus of your work there?*

A: I will be bringing my experience working on HIV and TB, including drug resistant strains of each, but I will also bring my perspective as a paediatrician. Antimicrobial resistance ties closely to paediatric health. For instance, neonatal and infant mortality can be significantly reduced by ensuring we have effective drugs for premature and vulnerable babies, particularly in low- and middle-income countries. In many ways, this role brings me back to my roots as a paediatrician, which is a great source of satisfaction. It feels like I’m coming home.

